# Band-like transport in solution-processed perylene diimide dianion films with high Hall mobility

**DOI:** 10.1093/nsr/nwae087

**Published:** 2024-03-08

**Authors:** Yanhua Jia, Qinglin Jiang, Hanlin Gan, Bohan Wang, Xiandong He, Jiadong Zhou, Zetong Ma, Jiang Zhang, Yuguang Ma

**Affiliations:** Institute of Polymer Optoelectronic Materials and Devices, State Key Laboratory of Luminescent Materials and Devices, South China University of Technology, Guangzhou 510640, China; Institute of Polymer Optoelectronic Materials and Devices, State Key Laboratory of Luminescent Materials and Devices, South China University of Technology, Guangzhou 510640, China; Institute of Polymer Optoelectronic Materials and Devices, State Key Laboratory of Luminescent Materials and Devices, South China University of Technology, Guangzhou 510640, China; Institute of Polymer Optoelectronic Materials and Devices, State Key Laboratory of Luminescent Materials and Devices, South China University of Technology, Guangzhou 510640, China; Institute of Polymer Optoelectronic Materials and Devices, State Key Laboratory of Luminescent Materials and Devices, South China University of Technology, Guangzhou 510640, China; Institute of Polymer Optoelectronic Materials and Devices, State Key Laboratory of Luminescent Materials and Devices, South China University of Technology, Guangzhou 510640, China; Institute of Polymer Optoelectronic Materials and Devices, State Key Laboratory of Luminescent Materials and Devices, South China University of Technology, Guangzhou 510640, China; Department of Physics, South China University of Technology, Guangzhou 510640, China; Institute of Polymer Optoelectronic Materials and Devices, State Key Laboratory of Luminescent Materials and Devices, South China University of Technology, Guangzhou 510640, China

**Keywords:** band-like transport, dianion aggregates, Hall mobility, solution-processed, perylene diimide

## Abstract

It is crucial to prepare high-mobility organic polycrystalline film through solution processing. However, the delocalized carrier transport of polycrystalline films in organic semiconductors has rarely been investigated through Hall-effect measurement. This study presents a strategy for building strong intermolecular interactions to fabricate solution-crystallized p-type perylene diimide (PDI) dianion films with a closer intermolecular π–π stacking distance of 3.25 Å. The highly delocalized carriers enable a competitive Hall mobility of 3 cm^2^ V^−1^ s^−1^, comparable to that of the reported high-mobility organic single crystals. The PDI dianion films exhibit a high electrical conductivity of 17 S cm^−1^ and typical band-like transport, as evidenced by the negative temperature linear coefficient of mobility proportional to *T*^−3/2^. This work demonstrates that, as the intermolecular π–π interactions become strong enough, they will display high mobility and conductivity, providing a new approach to developing high-mobility organic semiconductor materials.

## Teaser

This work presents solution-processed perylene diimide dianion films with strong intermolecular interaction, achieving band-like transport behavior with high Hall mobility in polycrystalline organic semiconductive film.

## INTRODUCTION

Low carrier mobility is the core problem that severely hinders the development and application of organic semiconductors [1,2]. Inorganic semiconductors are strong interaction systems in which atoms or ions are arranged in a regular periodic order to form a crystalline structure. Their transport mechanism can be understood through the band model. In contrast to inorganic semiconductors, the molecules in organic semiconductors are held together by weak intermolecular forces, such as van der Waals interaction and hydrogen bonds. The carrier transport properties of organic semiconductors depend significantly on large conjugated π structures with delocalized electrons in the molecule. However, it is challenging to achieve intermolecular charge delocalization in whole organic semiconductors as the charges are usually localized on each molecule. Thus, the transport of carriers mostly obeys the hopping mechanism at localized energy levels, resulting in low intrinsic carrier mobility (<10^−3^–10^−2^ cm^2^ V^−1^ s^−1^). Constructing strong intermolecular interactions is an effective strategy to improve the charge mobility of organic semiconductors.

The strong interaction facilitates orderly intermolecular π-stacking, enabling electron delocalization in organic aggregates and establishing channels for carrier transfer. The interactions between large and planar areas of two similar or identical π or π* frontier molecular orbitals were invoked to form π-stacked arrangements [3]. Intermolecular covalent π–π bonding-induced interaction has been indirectly demonstrated in stacked phenalenyl units along the chain due to the geometries of crystal packing [4]. Ion radicals with conjugated π structures (such as tetrathiafulvalene (TTF) and tetracyanoquinodimethane (TCNQ) anion radicals) have exhibited the propensity to form π-dimers [5–7]. Electron delocalization in π–π stacked dianion aggregates may yield impressively magnetic and conductive properties [8,9]. Our group recently reported that perylene diimide (PDI) radical aggregates exhibited unprecedented room-temperature ferromagnetism [10]. With a plane π-conjugate structure, the PDI dianion aggregates may be an ideal organic semiconductor for investigating charge delocalization between molecules. However, the structure and electrical transport properties of PDI dianion aggregates still lack investigation.

Efficient experimental tools are crucial for understanding the intrinsic properties of carrier transport in organic semiconductors. Organic field-effect transistor (OFET) devices are widely used to explore the correlation between the molecular structure of an organic semiconductor and its electronic properties [11–13]. However, OFET characterizations cannot measure the degree of carrier delocalization [14]. The Hall measurement is a typical way to understand carrier delocalization in semiconductors and obeys the Lorenz force that gives rise to the well-understood magnetic field effects [15]. The Hall effect has been successfully used to identify the fundamental properties of charge transport in single-crystal organic semiconductors [16–18]. Hall-effect measurements of organic semiconductor polycrystalline films are still rare due to their lower carrier mobility, resulting in a poor signal-to-noise ratio. This problem has been partly solved by applying gate-modulated Hall measurements in OFET devices. However, this also leads to the challenge of carrier trapping and scattering associated with interfacial charge transport [19]. Measuring the mobility of intrinsic carriers through a simple Hall-bar configuration without gate modulation is crucial for understanding the transport mechanisms of organic polycrystalline thin films.

In this work, PDI dianion films were fabricated through a solution-processing of the reduction and dissolution procedure in hydrazine hydrate. The X-ray diffraction patterns and high-resolution transmission electron microscopy images revealed π–π stacking with a closer π–π distance of 3.25 Å in the PDI dianion film. Hall measurements were conducted directly on the PDI polycrystalline film using the bottom contact of electrodes without gate modulation. The PDI dianion films exhibit p-type transporting characteristics with a Hall hole mobility of 3 cm^2^ V^−1^ s^−1^ at 150 K and a room-temperature electrical conductivity of 17 S cm^−1^. This outstanding performance was attributed to the high carrier delocalization, resulting in band-like transport behavior.

## RESULTS AND DISCUSSION

### Fabrication and composition of PDI dianion film

**Figure 1.**
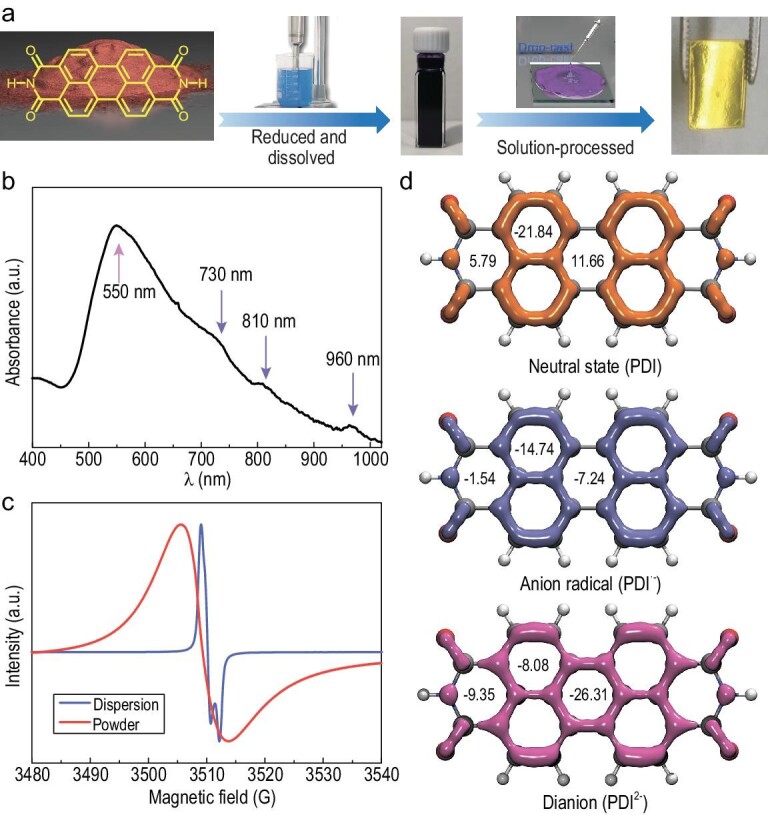
The composition of the PDI dianion aggregates. (a) The PDI dianion films were solution-processed by using the ultrasonic disrupter method. (b) The optical absorption spectrum of spin-coated PDI dianion films. (c) EPR spectra of the PDI dispersion and the dianion powder. (d) The NICS(1)_zz_ values and LOL-π isosurfaces of PDI, PDI^•−^ and PDI^2−^.

Raw PDI powder was reduced and dissolved in excess hydrazine hydrate to prepare for PDI dispersion by using an ultrasonic disrupter (Fig. 1a). The PDI film was easily solution-processed by drop-casting and displayed a metallic luster. It was prepared by spin-coating on a quartz substrate to measure the absorption spectrum in a glove box (O_2_ and H_2_O < 0.01 ppm). As shown in Fig. 1b, the spin-coated film exhibits one broad peak at ∼550 nm, along with weak peaks at 730, 810 and 960 nm. The broad peak is identified as PDI dianion (PDI^2−^) aggregates and the weak peaks are ascribed to the characteristic absorption of the radical anion (PDI^•−^) [20]. To further confirm the presence of the PDI^2−^ molecule and its aggregates, we investigated the concentration-dependent absorption spectra of PDI in N_2_H_4_·H_2_O (Fig. S1). The results suggest that the broad peak of the PDI film was identified as the PDI^2−^ aggregates. The broadening in absorption could be explained by the presence of intermolecular π-electron delocalization within the aggregates. Quantitative analysis (Figs S2 and S3) showed that the counter anion in the PDI dianion film was NH_4_^+^, which was introduced into the N_2_H_4_·H_2_O reducing process. According to Mulliken atomic charge analysis (Fig. S4), the charges of NH_4_^+^ and PDI^2−^ are 0.903 and –1.806, respectively. Hence, no charge transfer occurs in NH_4_^+^ and PDI^2−^. NH_4_^+^ acts as the counter anion in the PDI dianion film. Electron paramagnetic resonance (EPR) spectra were used to probe the formation of radical anions in the PDI dispersion and dianion powder (Fig. 1c). The asymmetric EPR of PDI dispersion indicated strong intermolecular π-electron delocalization along the π-stack. The anisotropic g-tensor is consistent with previously reported observations [21]. The PDI dispersion was evaporated to obtain the PDI dianion powder in the glove box. The EPR spectra of the PDI dianion powder exhibit a broadening peak-to-peak linewidth, indicating the presence of exchange coupling in the π-stacks [22]. The asymmetrical EPR characteristics of the powder are similar to the Dysonian line shape and are attributed to conduction electron spin resonance (CESR) [22,23]. The PDI dianion powder was ground to reduce its particle size and a symmetric line was subsequently observed (Fig. S5). Therefore, the unpaired electrons are delocalized throughout the PDI dianion aggregates.

Due to the planarity of PDI, PDI^•−^ and PDI^2−^, we used Multiwfn to identify the occupied canonical molecular orbitals (CMOs) with π characteristics and set the occupancy of other CMOs to zero. LOL-π was the localized orbital locator of electrons occupying the aforementioned π CMOs and was a real space function that was commonly used to visualize the delocalization range of π electrons [24,25]. The LOL-π maps [26,27] revealed the delocalization channel of the π electrons in PDI, PDI^•−^ and PDI^2−^. It was observed that the LOL-π isosurface over the entire perylene core was fully connected in PDI^2−^, while the isosurfaces between the two naphthalene rings were broken up in both PDI and PDI^•−^ (Fig. 1d). A stronger π conjugation in PDI^2−^ is inferred from this figure than in PDI and PDI^•−^. The zz component of nucleus-independent chemical shift (NICS) [28] values at points 1 Å above the ring centers has been widely used to characterize aromaticity and anti-aromaticity as quantitative aromaticity indexes. Negative NICS(1)_zz_ values indicate the presence of induced diatropic ring currents and aromaticity, while positive values denote paratropic ring currents and anti-aromaticity. The NICS(1)_zz_ values reveal apparent aromatic behavior for four of the six-membered rings of naphthalene moieties in PDI (–21.84 ppm), while its center and the hexahydric imide ring are nonaromatic (11.58 and 5.79 ppm, respectively). The molecule PDI^2−^ exhibits aromatic behavior in the naphthalene moieties (–8.08 ppm), the six-membered rings (–26.31 ppm) and the hexahydric imide rings (–9.35 ppm). Based on the above theoretical results, compared with PDI and PDI^•−^, PDI^2−^ exhibits a larger range delocalization of π electrons over the whole molecular area.

### Structural characterization of PDI dianion film

**Figure 2.**
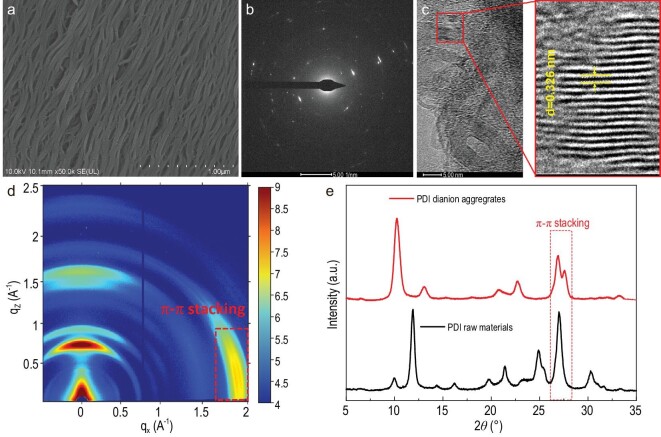
Structural characterization of the PDI dianion aggregates. (a) SEM image. (b) The corresponding SADE pattern. (c) The typical cryo-TEM image of the structure at high magnification. (d) GIWAXS pattern of the PDI dianion film. (e) XRD patterns of the PDI dianion aggregates and the PDI raw material.

Upon scanning electron microscopy (SEM), the nanowires appear densely packed and measured several microns in length and 42 nm in diameter (Fig. 2a). Selected-area electron diffraction (SADE) patterns display scattered diffraction patterns and smaller rings, indicating its polycrystalline structure in PDI dianion film (Fig. 2b). High-resolution transmission cryo-electron microscopy (Cyro-TEM) reveals a crystal lattice spacing of 3.26 Å, corresponding to the π–π stacking distance (Fig. 2c). The grazing incidence wide-angle X-ray scattering (GIWAXS) images show visible arcs of the diffracted signals, implying crystallite formation with an edge-on orientation (Fig. Figure S6 shows the 1D scattering profiles of the PDI dianion films. The new π–π stacking single that appeared at 1.95 Å^−1^ corresponds to a π–π stacking distance of 3.22 Å. Compared with our previous report [29], the closer π–π stacking distance was confirmed. Figure 2e shows the X-ray diffraction (XRD) patterns of the raw PDI materials and the dianion aggregates. The peak value of the raw materials, at 27.11°, indexed as (${\mathrm{12\bar{2}}}$) and corresponded to π–π stacking with a distance of 3.29 Å. The diffraction peaks in the PDI dianion aggregates at 27.42° corresponded to a π–π stacking distance of 3.25 Å (Fig. S7). Compared with PDI raw materials, the shorter π–π stacking distance is confirmed through the three characterization methods mentioned above. The electrostatic charge on the PDI molecules caused them to repel each other, increasing their distance. Hence, the closer spacing between the PDI dianion aggregates is unprecedented and involves other forces in the system that reduce the overall energy, such as pancake bonds [30]. Short multicenter interradical contact distances were observed, resulting in large transfer integrals and specific properties for pancake intermolecular π-bonds [31,32]. Theoretical calculations show that two orbitals exhibit the characteristics of pancake bonding in the central pair of the PDI dianion tetramer (Fig. S8).

### Electrical transport properties of PDI dianion film

**Figure 3.**
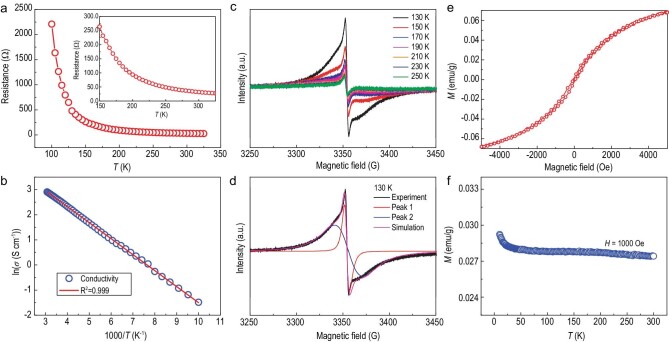
The transport properties and delocalized electron behavior of the PDI dianion films. (a) The temperature dependence of resistance. (b) The fitting curve of electrical conductivity versus the reciprocal temperature (150–325 K). (c) EPR spectra at various temperatures. (d) The simulation EPR spectra at 130 K. (e) The *M*–*H* hysteresis curve of the PDI dianion aggregates. (f) Temperature dependence of magnetic susceptibility at 1000 Oe.

The PDI dianion films exhibited an electrical conductivity of 17 S cm^−1^ at room temperature, which provided a remarkable advantage in doped organic small-molecule films. Table S1 summarizes the reported electrical conductivities of the organic doped small-molecule film since 2000. Transport properties of PDI dianion films were analysed by measuring temperature-dependent resistance (Fig. 3a). The resistance increased as the temperature decreased from 325 to 100 K, which is a typical semiconductor feature. Figure 3b shows the plot of ln *σ* versus 1/*T* according to the Arrhenius equation:


(1) \begin{eqnarray*}
\sigma = \mathrm{\it A exp}\!\left( { - \frac{{{{E}_a}}}{{{{k}_B}T}}} \right)
\end{eqnarray*}

where *k*_B_ is the Boltzmann constant, 0.0861733 meV K^−1^.

The plot (100–325 K) fits well with the model of thermal excitation and has an activation energy of *E*_a_ ≈ 48 meV. Compared with intrinsic semiconductors, the electrical conductivity of a doped semiconductor depends on the temperature and doping level [33]. The activation energy was likely associated with doping or structural defects [34,35]. The temperature dependence of EPR was analysed at between 130–250 K to understand the carrier transport behavior of PDI dianion aggregates. Figure 3c shows that the width, shape and intensity of the electron-spin resonance exhibit a distinct temperature dependence. The simulation EPR spectra are based on the overlap of two peaks (Fig. S9). Peak 1 belongs to the CESR signal and the peak intensity ratio remains constant at ∼1.2, which is lower than the 2.7 in metals [36]. As the temperature decreases, Peak 2 is significantly enhanced, resulting in a significant broadening of the experimental EPR absorption in Fig. 3d. The appearance of the broader peak suggests the correlation of spins, as observed in the Tetrakis(dimethylamino)ethylene-C_60_ and pyrolytic carbon [37,38]. As illustrated in the literature [39,40], two leading models for the broad EPR component are the conducting electrons coupled to defects and the random exchange model. The linear *M*–*H* curve of the PDI dianion aggregates reveals paramagnetic features (Fig. 3e). Figure 3f illustrates that the magnetic susceptibility was constant and temperature-independent throughout the temperature range (an upward tail was observed at <20 K), indicating the Pauli-like paramagnetism of the conduction electrons [41]. Thus, the PDI dianion aggregates exhibit semiconducting behavior with highly π-delocalized electrons.

**Figure 4.**
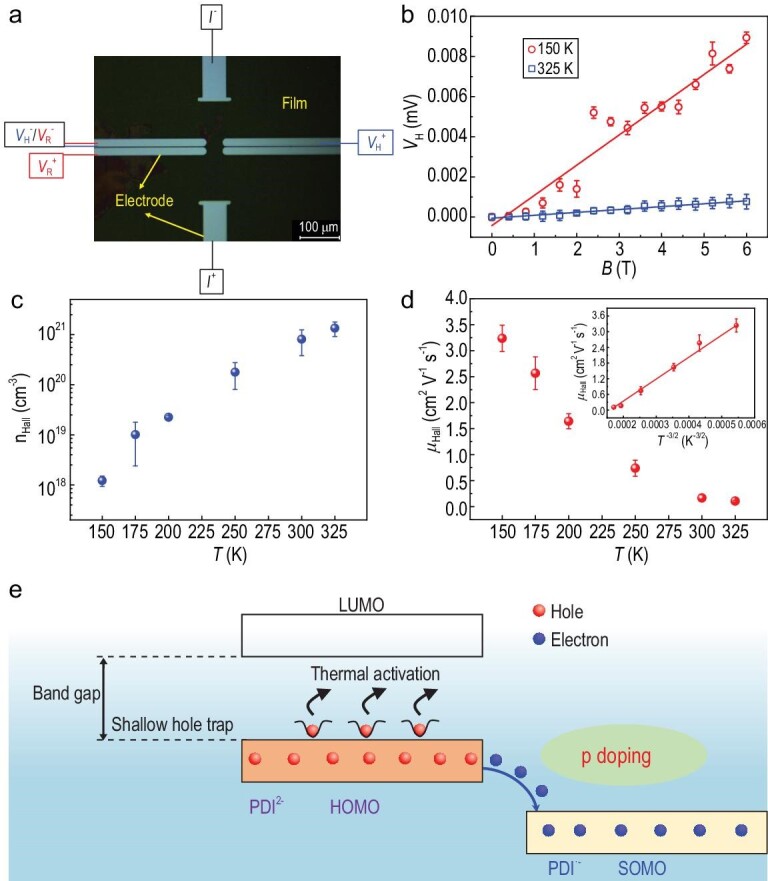
Hall measurements of the PDI dianion films. (a) Optical images of the Hall bar and the electrode configuration. (b) Profile of the Hall voltage (*V*_H_) with respect to magnetic fields at *T* = 325 and 150 K. (c) Carrier concentration versus temperature. (d) Hall mobility versus temperature. Inset: Hall mobility as a function of *T*^−3/2^. (e) The schematic diagram of the p-type band-like transport mechanism in the PDI dianion aggregates.

Six-bar Hall-effect measurements were conducted to investigate the carrier transport behavior of the PDI dianion film, which provides a standard characterization for measuring the extent of carrier delocalization. Figure 4a shows optical images of the Hall bar, while details of the electrode configuration are shown in Fig. S10. The Hall resistance (*R*_xy_) was measured in the range of temperatures from 150 to 325 K under magnetic fields (*B*) of 0–6 T. Figure 4b shows linear plots of the Hall voltage (*V*_H_) versus *B* at 150 and 325 K. The positive value of *V*_H_ indicates that carriers in the PDI dianion films are holes, which is consistent with the positive Seebeck coefficient (Table S2). The Hall coefficient (*R*_H_), carrier concentration and Hall mobility could be extracted from the linear curves (Fig. S11) and the results are summarized in Table S3. The carrier concentration increases with the increasing temperature (Fig. 4c). Figure S12 shows the fitting curve of the carrier concentration versus the reciprocal temperature (150–325 K) and reveals an activation energy (*E*_A_) of 165 meV in the PDI dianion film. Similar phenomena have been observed in doped inorganic semiconductors [33,42]. Conversely, the carrier mobility decreases (∼3 to ∼0.1 cm^2^ V^−1^ s^−1^) as the temperature is increased from 150 to 325 K (Fig. 4d). This phenomenon was representative of band-like transport and could be attributed to scattering processes induced by lattice phonons [43].

The Hall mobilities of the PDI dianion aggregates were found to be directly proportional to *T*^–^^3/2^, with a linear correlation coefficient of *R*^2^ = 0.956. This indicated that a band-like mechanism mainly dominated the transport properties in these aggregates. The PDI dianion aggregates with delocalized carriers exhibited strong electronic interactions, which induced band-like transport. Figure 4e illustrates that p-type transport behavior was observed in the PDI dianion film, where PDI^•−^ acted as a p-type dopant [29]. The holes were formed in the highest occupied molecular orbital (HOMO) of PDI^2−^ when the electrons in the HOMO of PDI^2−^ were activated (*E*_A_) and were transferred from the HOMO orbit of PDI^2−^ to the singly occupied molecular orbitals orbit of PDI^•−^. Structural defects in the films resulted in the capture of holes by shallow traps. As the temperature increases, the trapped holes gain energy (*E*_a_) and enter the HOMO of PDI^2−^, becoming free holes. Hence, the doping process and shallow hole traps determined the increase in carrier concentration.

Table 1 summarizes the electrical conductivity and Hall mobility of single-crystal, polycrystalline and polymer films based on Hall-effect measurements. The PDI dianion films exhibited a higher Hall mobility than single organic crystals and doped conjugated polymers. Compared with conductive polymers, small-molecular systems still have excellent potential for development, including batch-to-batch consistency and a comprehensive understanding of the transporting mechanism. Hence, this simple and inexpensive solution-processing method could be applied to develop high-mobility polycrystalline films. Table S4 summarizes the carrier mobility of PDI analogous crystals characterized by the OFET device. Comparing the interfacial mobility based on OFET and bulk transport based on Hall-effect measurement is not inappropriate. Moreover, unified standards are required for Hall and OFET mobility, including temperature, exposure conditions, channel length and applied voltage. Each parameter corresponds to a specific device structure. In our work, we extracted the Hall mobility of an ungated PDI dianion film through a simple device, which provided a better understanding of the intrinsic carrier transport mechanism.

**Table 1.** Summary of electrical conductivities and Hall mobilities of organic semiconductors.

Materials	Categories	Fabrication method	Conductivity (S cm^−1^)	Mobility (cm^2^ V^−1^ s^−1^)	Reference
PDI	Small-molecular polycrystalline film	Solution-processed	17	3 (150 K)^a^	This work
DNTT		Physical vapor transport	NA	1.1 (160 K)^b^	[44]
Pentacene		Physical vapor transport	NA	1.5 (280 K)^b^	[45]
C_10_-DNTT		Solution-processed	8 × 10^−6^ (*V*_G_ = –100 V)	12 cm^2^/V (300 K)^b^	[46]
C_8_-BTBT		Solution-processed	4 × 10^−6^ (*V*_G_ = –120 V)	10 cm^2^/V (300 K)^b^	[46]

Rubrene	Single-crystal	Physical vapor transport	NA	11^a^	[19]
DNTT		Physical vapor transport	NA	3 (280 K)^b^	[44]
Pentacene		Physical vapor transport	NA	2 (280 K)^b^	[45]
BTQBT		Recrystallization/sublimation	0.83 × 10^−6^	4^b^	[18]
PEDOT : TfO	Conductive polymer film	Spin-coated	1987	3.7 (300 K)/1.5 (32 K)^c^	[47]
PEDOT : PSS		Spin-coated	640	0.5 (300 K)/0.02 (32 K)^c^	[47]
PBTT-C16		Spin-coated	∼100	1.5^a^	[48]
FBDPPV-OEG		Spin-coated	0.8/39	0.14/1.3 × 10^−3^^c^	[49]
PDPP-4T		Drop-casting	∼10	0.05–0.3^d^	[50]
P3HT		Drop-casting	∼25	0.2–0.3^d^	[50]
DDB-F72-P3HT		Solution-processed	12.8	0.1^c^	[51]
FT4NQ-P3HT		Solution-processed	1.5	0.02^c^	[51]

a Hall effect. ^b^Hall effect in OFET. ^c^AC Hall measurement. ^d^AC Hall measurement in OFET.

## CONCLUSION

In conclusion, we have demonstrated band-like transport in solution-processed p-type PDI dianion films with a high Hall mobility. The drop-casted PDI polycrystalline films exhibit a closed intermolecular π–π distance of 3.25 Å. The conduction electron spin resonance and temperature-independent Hall mobility verify the delocalized carrier transport. Mobility measurement of ungated films through a simple Hall-bar configuration shows a competitive electrical conductivity of 17 S cm^−1^ and p-type transporting characteristics, with a Hall hole mobility of 3 cm^2^ V^−1^ s^−1^. The negative temperature linear coefficient of mobility is proportional to *T*^−3/2^, suggesting a typical band-like transport mechanism. These results indicate that constructing strong intermolecular interactions can be an effective strategy for developing high-mobility organic semiconductor films.

## METHODS

### Preparation of PDI dianion films

The 55 mg of PDI powder was added to 5 mL of N_2_H_4_•H_2_O and then dispersed using an ultrasonic cell crusher with an ultrasonic power of 540 W and 0°C applied for 15 minutes. This processing method is optimized, differing from our previously reported one [29], in which a PDI dianion film was fabricated through an autoclave and heated at 140°C for 24 h. Then, 11 mg mL^−1^ of PDI-N_2_H_4_•H_2_O dispersions were drop-casted on cleaned substrates in the glove box. Finally, the as-prepared films were dried in a glove box at 50°C for 12 h. The PDI-N_2_H_4_•H_2_O dispersions were spin-coated on a cleaned quartz substrate to measure the absorption spectrum.

## Supplementary Material

nwae087_Supplemental_File
