# Supplementary material for: Band-like transport in solution-processed perylene diimide dianion films with high Hall mobility
Source: Natl Sci Rev. 2024 Mar 8;11(5):nwae087. doi: 10.1093/nsr/nwae087 (PMC11008685; doi:10.1093/nsr/nwae087)
Supplement: nwae087_Supplemental_File [file nwae087_supplemental_file.pdf]

Supplementary Data

Band-like transport in solution-processed perylene diimide dianion films with high Hall mobility

Yanhua Jia[1], Qinglin Jiang[1, *], Hanlin Gan[1], Bohan Wang[1], Xiandong He[1], Jiadong Zhou[1], Zetong Ma[1], Jiang Zhang[2, *] and Yuguang Ma[1, *]

[1]Institute of Polymer Optoelectronic Materials and Devices, State Key Laboratory of Luminescent Materials and Devices, South China University of Technology, Guangzhou 510640, China

[2]Department of Physics, South China University of Technology, Guangzhou 510640, China

[*]**Corresponding authors.** E-mail: jiangql@scut.edu.cn; jonney@scut.edu.cn; ygma@scut.edu.cn.

Table of Contents

Materials......................………………………………………….....................…..………..…....2

Computational Details……........………...……...……........................……..……………..………......…..4

Figures and Tables………………………………………………....................………………......5

References……………………………….…………………….…......................…..……………21

Materials

The 3,4,9,10-Perylenetetracarboxylic diimide PDI (98%) was purchased from *J & K Co., Ltd*. Hydrazine hydrate (98% in water, $N_2H_4 \cdot H_2O$) was obtained from Shanghai Aladdin Bio-Chem Technology Co., Ltd.

Characterization

The structure of the PDI film was characterized by using XRD (Rigaku SmartLab) with Cu Ka (k=0.15406 nm) radiation. The UV-vis-NIR characterization was performed on a SHIMADZU UV-3600 spectrophotometer (Kyoto, Japan). The EPR spectra were recorded on a Bruker E500 EPR spectrometer (300 K, 9.854 GHz, X-band, Karlsruhe, Germany). The microwave power was 6.325 mW, and the width of the sweep magnetic field ranged from 3400 to 3600 G. The modulation frequency was 100 kHz, and its amplitude was 1 G.

Morphology was characterized by using a field-emission scanning electron microscope (ZEISS, Oberkochen, Germany) at room temperature. The high-resolution transmission electron microscopy (HRTEM) and selected-area electron diffraction (SADE) were obtained by using a Talos F200 transmission electron microscope with a Ceta cameral. The working temperature was 96 K. The GIWAXS patterns were acquired by using a Xeuss 2.0 WAXS system (Xenocs, Sassenage, France) with a PILATUS3 R 1 M detector.

Magnetization was measured using Quantum Design PPMS Evercool II with a vibrating sample magnetometer over the 10-300 K temperature range. The Hall resistivity was measured by a six-contact Hall bar device by using the Quantum Design PPMS Evercool II with the Electrical Transport Option.

The film was deposited on a cleaned glass substrate enclosed in a circular copper holder with a thermocouple. A standard four-point probe technique with a Keithley 2700 and 2401 system (Keithley, Cleveland, OH, USA) was used to measure electrical conductivity and the Seebeck coefficient. The thickness of the film was characterized by a Dektak 150 surface profiler (Veeco Ins., USA).

Computational Details

The structures PDI, $PDI^{\bullet -}$, and PDI^{2-} were optimized by M05-2X functional [1] with 6-31+G(d) basis set [2, 3] for all atoms. Harmonic vibration frequency calculations were performed for all stationary points to confirm them as local minima. The $NICS(1)_{zz}$ values [4-6] were computed by employing the gauge-independent atomic orbital (GIAO) method. All the above calculations were accomplished by using the Gaussian 16 suite of programs [7]. The LOL-π isosurfaces [8, 9] based on canonical molecular orbitals were generated using Multiwfn 3.8(dev) software [10], and the isosurface map was produced using VMD (version 1.9.3) [11] software.

Figures and Tables

1. PDI^{2-} absorption spectrum in solution

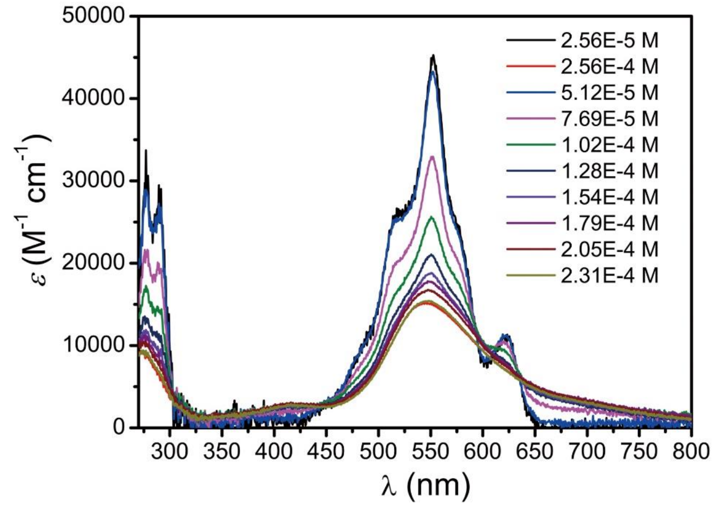

Figure S1. The concentration-dependent UV-Vis absorption spectra of PDI in $N_2H_4 \cdot H_2O$.

The PDI^{2-} molecule (2.56×10^{-5} M) exhibited characteristic peaks at 550 and 620 nm, with two shoulders at approximately 517 and 572 nm, similar to diimide radical anions reported by Wasielewski *et al*. [12] With increasing concentration, the peaks at 519, 572, and 622 nm weakened and disappeared, while the main peak at 550 nm had a slight red shift (5 nm). Hence, the absorption peak of PDI^{2-} aggregates is located at 550 nm (from 400 to 700 nm).

2. The X-ray photoelectron spectroscopy (XPS) of PDI dianion films

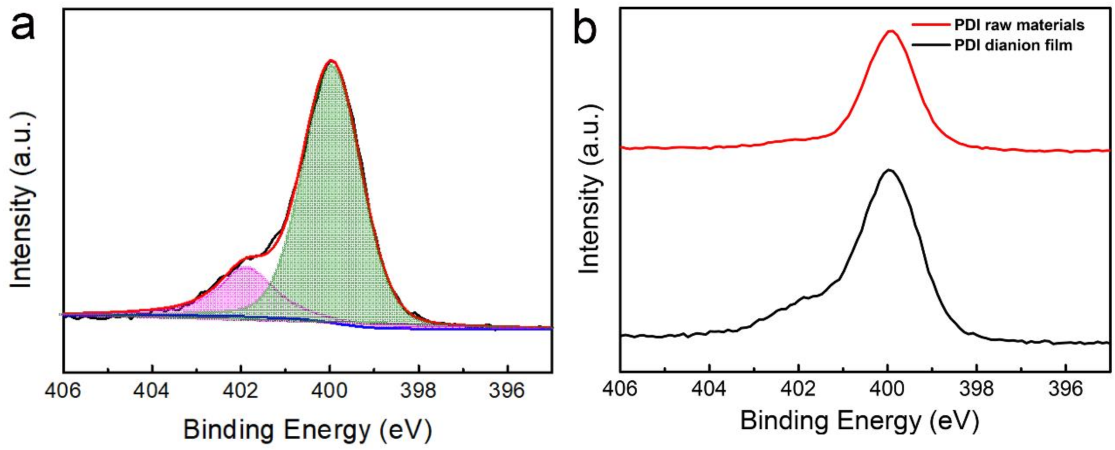

Figure S2. (a) The high-resolution XPS spectrum of N1s atom of PDI dianion film. (b) The high-resolution XPS spectrum of N1s atom of PDI raw materials and PDI dianion film.

XPS analysis of the N 1s peak from the PDI dianion film shows a broadened spectrum with two component peaks (Figure S2a). The peak at 399.9 eV was assigned to N-C=O chemical bindings [13]. The peak at higher binding energy (401.8 eV) is assigned to positively charged N atoms of quaternary ammonium (NH_4^+) cations [14]. Figure S2b shows the XPS narrow scan (399.9 eV) for the N 1s peak of PDI raw materials as a control experiment. It exhibits only one peak, indicating only one form of N in PDI raw materials.

3. Ion chromatography

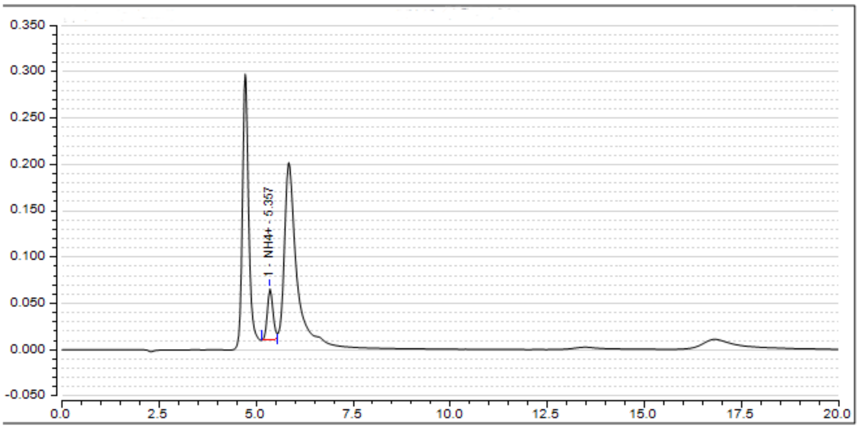

Figure S3. NH_4^+ detection by ion chromatograph in PDI dianion film.

According to JIS K0127-2013 (General Rules for Ion Chromatography), the NH_4^+ was confirmed by an ion chromatograph (ICS-1600).

4. Mulliken atomic charge analysis of $(NH_4)_2PDI$

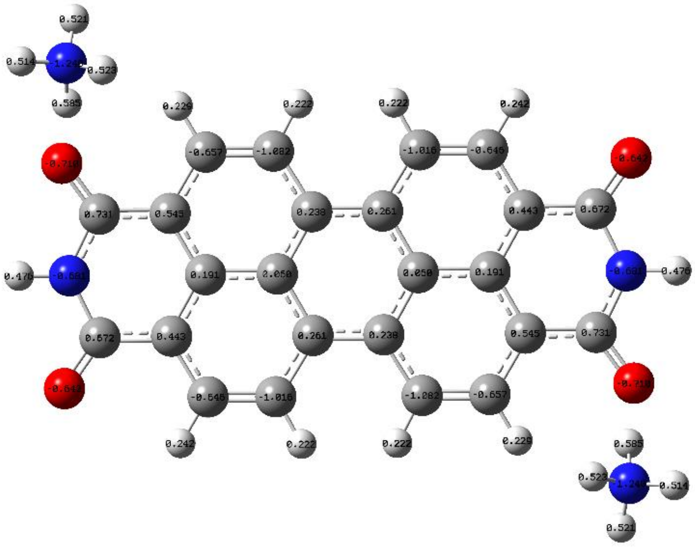

Figure S4. The Mulliken atomic charge analysis of $(NH_4)_2PDI$.

According to Mulliken atomic charge analysis, the charge of NH_4^+ and PDI^{2-} is 0.903 and -1.806, respectively.

5. Symmetric EPR characteristics

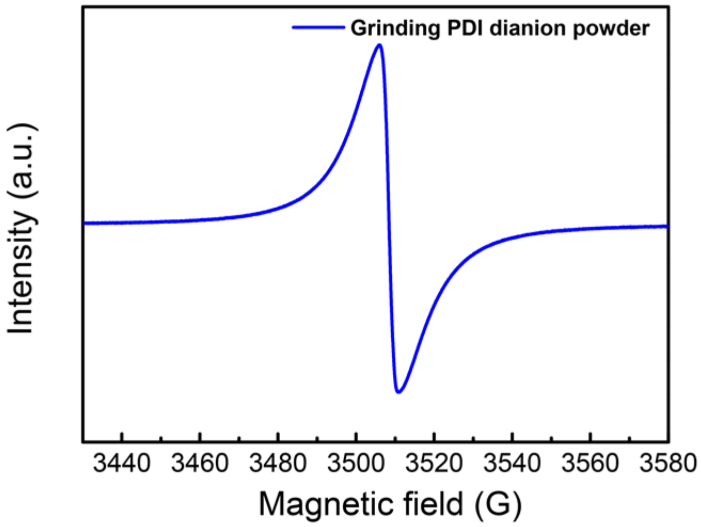

Figure S5. EPR spectra of PDI dianion powder with grinding.

6. GIWAXS comparison of PDI dianion film with different solution-processed methods.

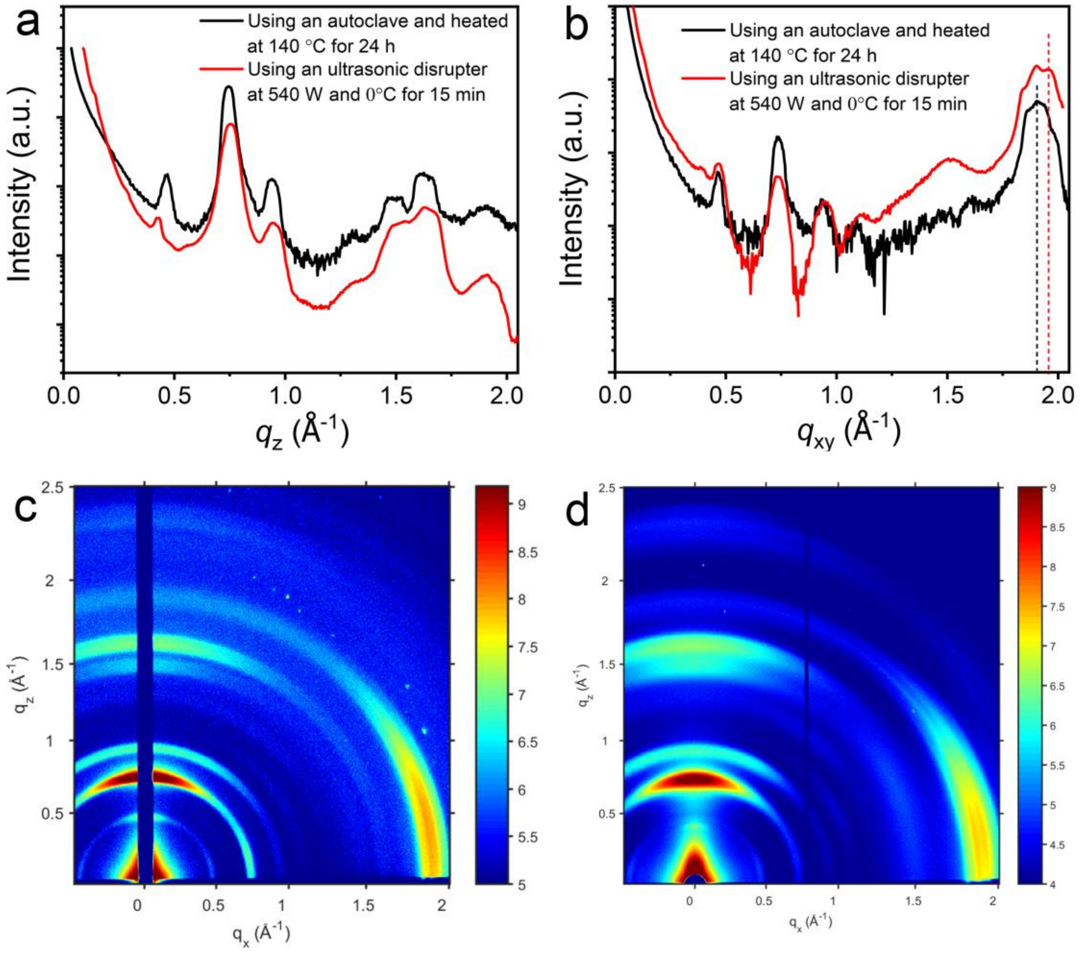

Figure S6. The direct comparison of 1D (a) out-of-plane (q_z) and (b) in-plane (q_{xy}) profiles. The 2D GIWAXS pattern with different solution-processed methods: (c) using an autoclave and heated at 140 ℃ for 24 h, and (d) using an ultrasonic disrupter at 540 W and 0 ℃ for 15 min.

The new π-π stacking single appeared at 1.95 Å in the in-plane (q_{xy}) direction, and the calculated distance was 3.22 Å. The 2D GIWAXS pattern demonstrated that different processing methods affect the aggregation structures.

7. XRD of PDI dianion aggregates

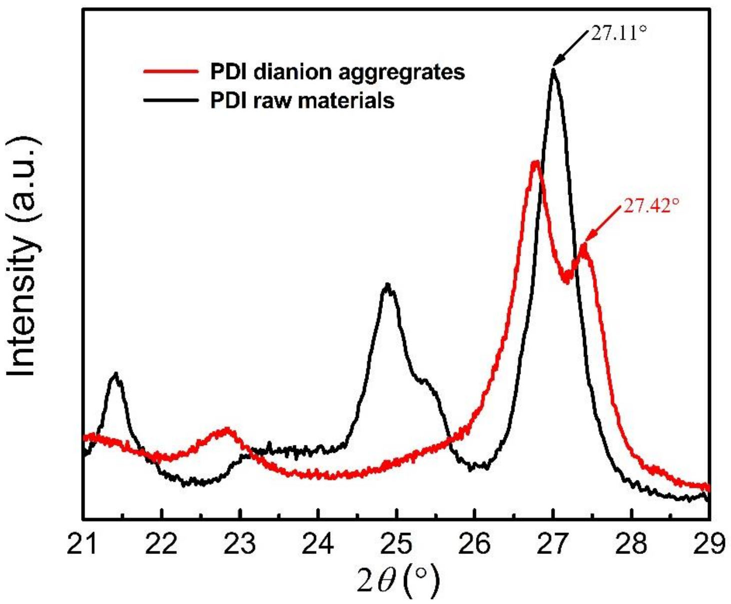

Figure S7. The changes of diffraction peak of PDI dianion aggregates.

The diffraction peaks of PDI dianion aggregates exhibited remarkable changes. According to Bragg's equation:

$$2\mathrm{d}\sin\theta = \mathrm{n}\lambda$$

λ=0.15406 nm, n=1, d is stacking distance;

The diffraction peak of PDI raw materials at 27.11 °corresponds to the stacking distance of 3.29 Å. The diffraction peak of PDI dianion aggregates shifted to higher angles of 27.42 °, corresponding to the stacking distance of 3.25 Å. As the peaks at 2θ of around 27 °originated from the diffracted signals of stacking π planes, the splitting peaks in anionic PDI related to that in the raw one could be attributed to the two kinds of π-stacked modes caused by the asymmetrical displacement of adjacent molecular scaffolds, leading to the changes of the π-distances.

8. The pancake orbital of PDI dianion tetramer

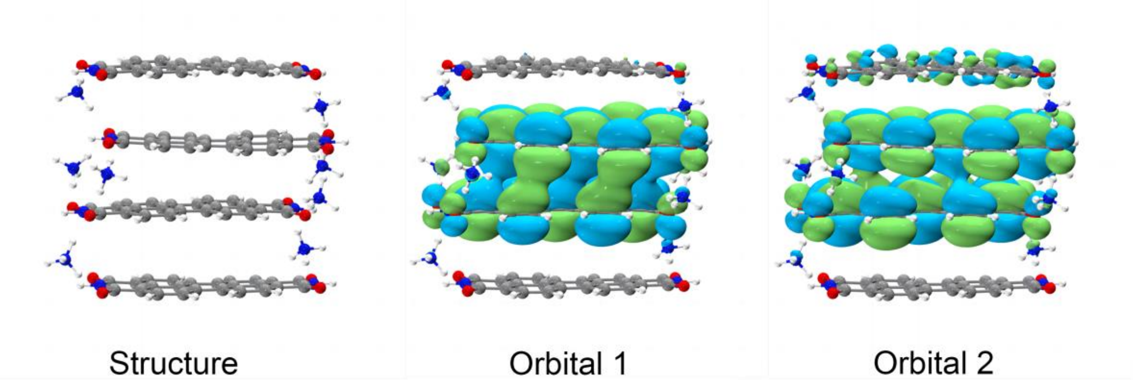

Figure S8. The structure and pancake bonding orbitals of PDI dianion tetramer.

A tetramer model was optimized at the theoretical level M05-2X/6-31G* to model the stacking structures in the condensed state. Two orbitals exhibit the characteristics of pancake bonding in the central pair of the PDI dianion tetramer.

9. The conductivity of doped organic small-molecular

Table S1. The conductivity of doped organic small-molecule film.

Years	Molecule	Conductivity (S cm^{-1})	*Ref.*
2023	PDI	17	This work
2021	NDI	0.11	[15]
2020	4Cl-PDI	0.18	[16]
2020	QTICN	0.34	[17]
2020	PTEG-2	13	[18]
2019	2DQQT	14	[19]
2019	2DQPT	2.85	[19]
2019	2DQBT	1.4×10^{-4}	[19]
2019	2DQQT-Se	0.29	[20]
2019	2DQQT-S	8.0×10^{-3}	[20]
2017	A-DCV-DPPTT	5.3	[21]
2017	D-DCM-DPPTT	0.1	[21]
2017	2DQTT	1.1	[22]
2016	Bis-HFI-NTCDI	9.4×10^{-5}	[23]
2016	ZnPc	1.1×10^{-2}	[23]
2014	PDI-3	0.5	[24]
2013	FPI	2.0×10^{-2}	[25]
2000	NTCDA	1×10^{-5}	[26]

10. The EPR simulation curve at different temperatures

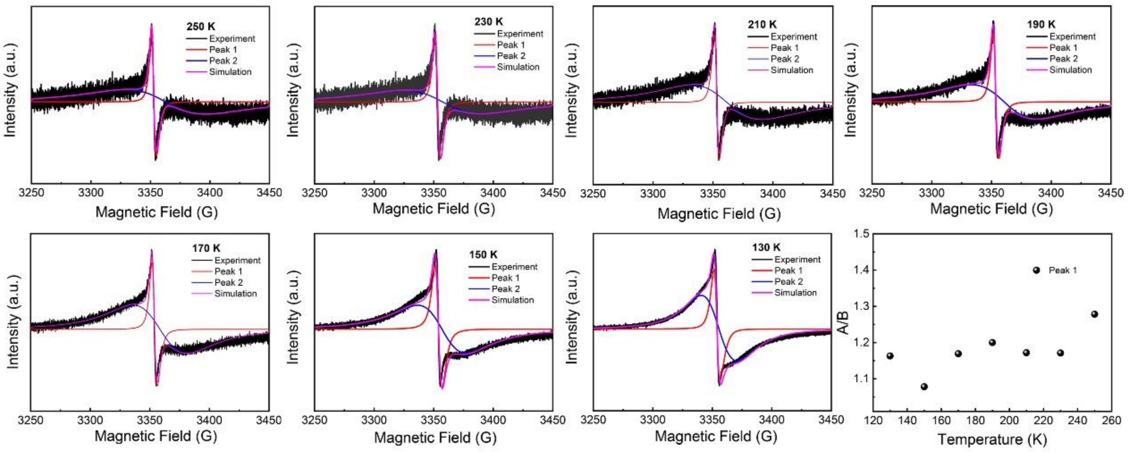

Figure S9. The simulation EPR spectra at different temperatures.

11. Hall effect measurement of PDI dianion film

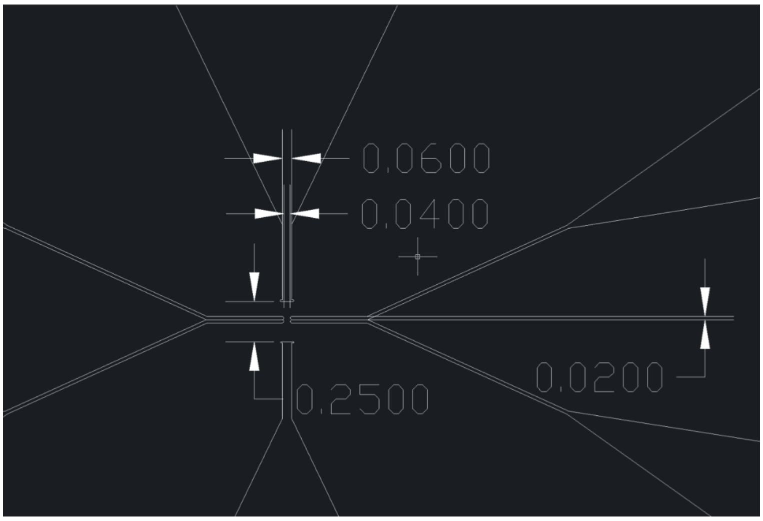

Figure S10. Schematic of the Hall bar and dimension of the electrode.

A bottom contact geometry was chosen for the Hall bar to avoid damage to the PDI dianion film owing to the aggressive solvents used in the photolithography process. The distance between horizontal electrodes was 0.002 mm, and between vertical electrodes was 0.25 mm.

12. Thermoelectric performance of PDI dianion film

Table S2. The electrical conductivity and Seebeck coefficient of the PDI dianion film.

Sample	Thickness (nm)	Conductivity (S cm^{-1})	Seebeck coefficient (μV K^{-1})
1	2956	16.02	13.33
2	3113	16.32	11.24
3	3432	19.37	10.59
Average	3167±242	17.25+1.84	11.72±1.43

The electronic conductivity (σ) and Seebeck coefficient (S) of the PDI dianion film were measured to investigate its charge transport properties. The PDI film exhibited a high electrical conductivity of 17 S cm^{-1} and a Seebeck coefficient of 12 μV K^{-1}, exhibiting p-type semiconductor characteristics.[27]

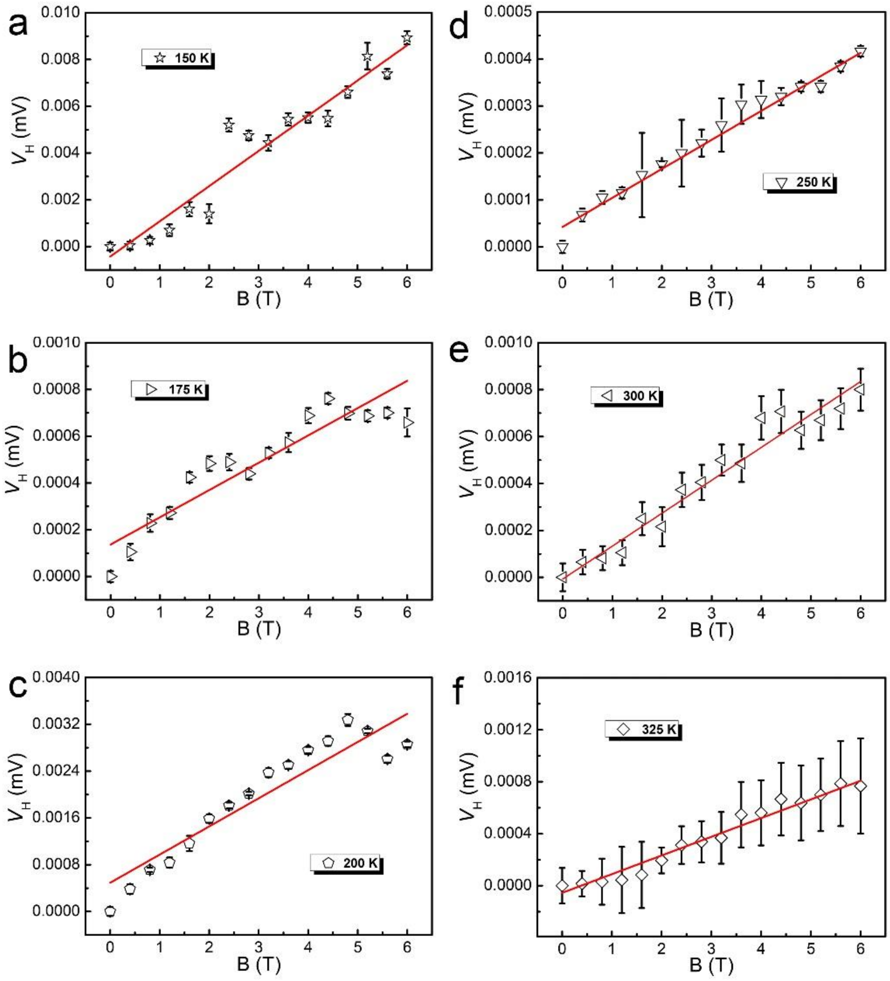

Figure S11. The dependence of the V_{Hall} of the PDI dianion films on the magnetic field at different temperatures.

The red line is a linear fit, and the Hall coefficient (R_H) was extracted from its slope. The error bars of carrier concentration and Hall mobility originated from uncertainty in the value of V_{Hall} and represented one standard deviation. Hall carrier concentration $n_{Hall} = (e \cdot R_H)^{-1}$, and Hall mobility at $\mu_{Hall} = \sigma \cdot R_H$ were derived from the usual procedure of Hall effect measurements.

Table S3. The Hall coefficients of PDI dianion aggregates at different temperatures.

Temperature (*K*)	Resistance (*Ω*)	R_H (cm^3 C^{-1})	R^2	Carrier type
325	10.22485	0.0038±0.0025	0.922	p-type
300	11.69527	0.0056±0.0048	0.960	p-type
250	18.19364	0.025±0.022	0.976	p-type
200	38.06646	0.25±0.061	0.992	p-type
175	96.15152	0.40±0.21	0.828	p-type
150	358.1212	4.37±1.56	0.895	p-type

13. The fitting curve of carrier concentration

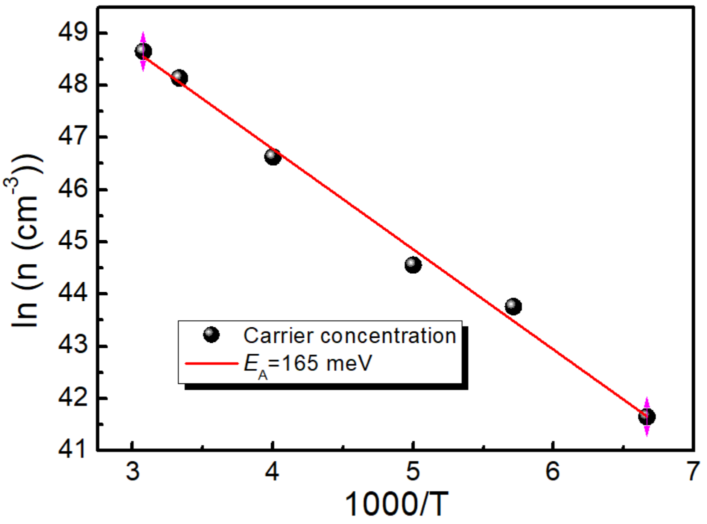

Figure S12. The fitting curve of carrier concentration versus the reciprocal temperature (150–325 K).

14. The mobility of PDI analogues

Table S4. The mobility of PDI analogues single crystal based on OFET device.

Molecule	Aggregation	Transport model	Mobility ($cm^2\ V^{-1}\ s^{-1}$)	*Ref.*
PDIF-CN_2	Single-crystal	Band-like	0.8	[28]
PDI8-CN_2	Single-crystal	Thermal activated	0.18	[28]
PDIF-CN_2	Single-crystal	Band-like	10.8	[29]
PDI3F/PDI5F	Single-crystal	Band-like	c.a. 5	[30]

References

1. Zhao Y, Schultz NE, Truhlar DG. Design of density functionals by combining the method of constraint satisfaction with parametrization for thermochemistry, thermochemical kinetics, and noncovalent interactions. *J Chem Theory Comput* 2006; **2:** 364-82.
2. Neese F, Wennmohs F, Becker U *et al*. The ORCA quantum chemistry program package. *J Chem Phys* 2020; **152:** 224108.
3. Clark T, Chandrasekhar J, Spitznagel GW *et al.* Efficient diffuse function-augmented basis sets for anion calculations. III. The 3-21+G basis set for first-row elements, Li-F. *J Comput Chem* 1983; **4:** 294-301.
4. Schleyer PvR, Maerker C, Dransfeld A *et al*. Nucleus-independent chemical shifts: a simple and efficient aromaticity probe. *J Am Chem Soc* 1996; **118:** 6317.
5. Chen Z, Wannere CS, Corminboeuf C *et al.* Nucleus-independent chemical shifts (NICS) as an aromaticity criterion. *Chem Rev* 2005; **105:** 3842-88.
6. Fallah-Bagher-Shaidaei H, Wannere CS, Corminboeuf C *et al.* Which NICS aromaticity index for planar π rings is best ? *Org Lett* 2006; **8:** 863-6.
7. Gaussian 16 Revision C.01, Gaussian, Inc., Wallingford CT; 2016.
8. Schmider HL, Becke AD. Chemical content of the kinetic energy density. *J Mol Struc-Theochem* 2000; **527:** 51-61.
9. Lu T, Chen Q. A simple method of identifying π orbitals for non-planar systems and a protocol of studying π electronic structure. *Theor Chem Acc* 2020; **139:** 25.
10. Lu T, Chen F. Multiwfn: a multifunctional wavefunction analyzer. *J Comput Chem* 2012; **33:** 580-92.
11. Humphrey W, Dalke A, Schulten K. VMD: Visual molecular dynamics. *J Molec Graphics*

1996; **14:** 33.

12. Gosztola D, Niemczyk MP, Svec W *et al.* Excited doublet states of electrochemically generated aromatic imide and diimide radical anions. *J Phys Chem A* 2000; **104:** 6545-51.
13. Bourbigot S, Bras ML, Gengembre L *et al.* XPS study of an intumescent coating application to the ammonium polyphosphate/pentaerythritol fire-retardant system. *Appl Surf Sci* 1994; **81:** 299-307.
14. Hojati-Talemi P, Evans D, Fabretto M. Extending the utility of conducting polymers through chemisorption of nucleophiles. *Chem Mater* 2013; **25:** 1837–41.
15. Zhao D, Jiang Q, Jia Y *et al.* High performance n-type thermoelectric material based on naphthalenediimide radical anions. *Mater Today Energy* 2021; **21:** 100710.
16. Jiang Q, Sun H, Zhao D *et al.* High thermoelectric performance in n-type perylene bisimide induced by the Soret effect. *Adv Mater* 2020; **32:** 2002752.
17. Yang K, Zhang X, Harbuzaru A *et al.* Stable organic diradicals based on fused quinoidal oligothiophene imides with high electrical conductivity. *J Am Chem Soc* 2020; **142:** 4329-40.
18. Liu J, Bas vdZ, Alessandri R *et al.* N-type organic thermoelectrics: demonstration of *ZT* > 0.3. *Nat Commun* 2020; **11:** 5694.
19. Yuan D, Huang D, Rivero S *et al.* Cholesteric aggregation at the quinoidal-to-diradical border enabled stable n-doped conductor. *Chem* 2019; **5:** 964-76.
20. Yuan D, Guo Y, Zeng Y *et al.* Air-stable n-type thermoelectric materials enabled by organic diradicaloids. *Angew Chem Int Ed* 2019; **58:** 4958-62.
21. Huang D, Yao H, Cui Y *et al.* Conjugated-backbone effect of organic small molecules for n-type thermoelectric materials with *ZT* over 0.2. *J Am Chem Soc* 2017; **139:** 13013-23.
22. Yuan D, Huang D, Zhang C *et al.* Efficient solution-processed n-type small-molecule

thermoelectric materials achieved by precisely regulating energy level of organic dopants. *ACS Appl Mater Interfaces* 2017; **9:** 28795-801.

23. Tietze ML, Rose BD, Schwarze M *et al.* Passivation of molecular n-doping: exploring the limits of air stability. *Adv Funct Mater* 2016; **26:** 3730-7.
24. Russ B, Robb MJ, Brunetti FG *et al*. Power factor enhancement in solution-processed organic n-type thermoelectrics through molecular design. *Adv Mater* 2014; **26:** 3473-7.
25. Li CZ, Chueh CC, Ding F *et al.* Doping of fullerenes via anion-induced electron transfer and its implication for surfactant facilitated high performance polymer solar cells. *Adv Mater* 2013; **25:** 4425-30.
26. Nollau A, Pfeiffer M, Fritz T *et al.* Controlled n-type doping of a molecular organic semiconductor: naphthalenetetracarboxylic dianhydride (NTCDA) doped with bis(ethylenedithio)-tetrathiafulvalene (BEDT-TTF). *J Appl Phys* 2000; **87:** 4340-3.
27. Zhang W, Jiang Q, Zhou J *et al.* Preparation and electronic characteristics of anionic perylene bisimide films. *Sci China Chem* 2017; **60:** 1334-9.
28. Stoeckel MA, Olivier Y, Gobbi M *et al*. Analysis of external and internal disorder to understand band-like transport in n-type organic semiconductors. *Adv Mater* 2021, **3:** e2007870.
29. Minder NA, Ono S, Chen Z *et al*. Band-like electron transport in organic transistors and implication of the molecular structure for performance optimization. *Adv Mater* 2012, **24:** 503-8.
30. Minder NA, Lu S, Fratini S *et al*. Tailoring the molecular structure to suppress extrinsic disorder in organic transistors. *Adv Mater* 2014, **26:** 1254-60.
